# 

**Published:** 1892-11-12

**Authors:** 


### CONTENTS.

PAGE

**New Medicine for the New Times** - - - - 97

**Passing Topics.**—The Schoolmaster very much Abroad - - 98

**Medical History from the Earliest Times.** By E. T. Withington, M.A., M.B.Oxon. XXXI.—Arabic Medicine. (4) The Western Caliphate - - - - - - - - - 99

**Everybody's Page** - - - - - - - - - - - 100

**The Doctor's Armchair.**—Mind, Matter, and Medicine - - 101

**The Attitude of Great Writers Towards Disease.** XV. —The Sick in Utopia - - - - - - - - - - 102

**Annotations.**—Where are the Glasgow Newspapers?—A Clearing-house for the Unemployed—Oxford Doings Extraordinary - - 103

**Notes and News!** - - - - - - - - - - - 104

**Around the Hospitals—Scraps and Gleanings** - - - 112

**The Student of Medicine.**—Notes from the Schools—Vacancies-

**The Hospital Clinic—**

Paddington Green Children's Hospital.—The Feeding of Infants and Children. I.—Health - - - - - - 105

Queen's Hospital, Birmingham.—Treatment of Chronic Ulcers of the Leg - - - - - - - - - - - - 106

Northampton General Infirmary.—The Choice of an Antiseptic - - - - - - - - - - - - - 106

**Aix-la Chapelle and its Waters** - - - - - - - 108

**The Institutional Workshop—**

Within the Hospitals.—Sheffield General Infirmary - - - 109

Practical Departments.—III. Children's Cots - - - - 110

Hospital Worthies. — Dr. John Charles Steele, M.D., L.R.C.P.Edin., F.F.P.S.Glasgow - - - - - - - 111

Practitioner's Bookshelf—Dissections Illustrated - - - 112

### EXTRA SUPPLEMENT.—THE NURSING MIRROR.

**En Passant.**—The R. B. N. A.—Friedenheim—Short Items—Windsor Nurses—The Endowed Bed for a Sick Nurse—Christmas Parcels and Competitions - - - - - xxxvii

**Lectures for Asylum Attendants.** By William Harding, M.B.—VII. Management of Patients - - - - - xxxviii

**Everybody's Opinion.**—A Nurse's Career—Lectures on Sick Nursing and Domestic Economy - - - - - - - xxxix

**The Registration of Nurses.**—The R.B.N.A. Scheme Rejected by the Lords' Committee - - - - - - xxxix

**Training in Massage** - - - - - - - - - - - xl

**The Workhouse Infirmary Nursing Association** - xl

**Death in Our Ranks** - - - - - - - - - - xli

**Appointments** - - - - - - - - - - - - xli

**Notes and Queries** - - - - - - - - - - xli

**For Reading to the Sick.**—Consolation - - - - - xli

**Some Australian Experiences** - - - - - - - xlii

**Examination Questions** - - - - - - - - - xlii

**Where to Go** - - - - - - - - - - - - - xlii

